# Learning interventions in the WHO Eastern Mediterranean region: supporting Member States to get prepared for better response to health emergencies in the region

**DOI:** 10.3389/fpubh.2024.1441223

**Published:** 2024-09-12

**Authors:** Mohamed Elhakim, Samar Dimachkie Hammoud, Sabri Gmach, Jehan Albadri, Heba Mahrous, Fatima Arifi, Nisreen Abdel Latif, Safaa Moussa, Huda Anan, Asma Saidouni, Ramy Mohamed Ghazy, Amgad Elkholy, Hammam Elsakka, Dalia Samhouri

**Affiliations:** ^1^Country Health Emergency Preparedness and International Health Regulations, WHO Health Emergencies Program, World Health Organization Eastern Mediterranean Regional Office, Cairo, Egypt; ^2^Family and Community Medicine, College of Medicine, King Khalid University, Abha, Saudi Arabia; ^3^Tropical Health Department, High Institute of Public Health, Alexandria University, Alexandria, Egypt

**Keywords:** Eastern Mediterranean region, strategic risk assessment, health emergency preparedness, international health regulations, risk communication and community engagement, chemical biological and radio nuclear events

## Abstract

**Background:**

The Eastern Mediterranean Region (EMR) faces numerous public health risks caused by biological, chemical, man-made, and natural hazards. This manuscript aimed to assess the multifaceted interventions and strategies used to strengthen the EMR’s preparedness capacities to respond properly to current and upcoming health emergencies.

**Objective:**

To address these challenges, it is crucial to implement comprehensive and robust strategic risk assessments and health emergency preparedness frameworks. The World Health Organization (WHO) takes a risk-based approach, emphasizing the significance of all-hazards emergency management and the creation of national health risk profiles using the Strategic Toolkit for Assessing Risk (STAR). Furthermore, the International Health Regulations (IHR) Monitoring and Evaluation Framework (MEF) ensures continuous learning and capacity building among Member States, enhancing their ability to manage health emergencies effectively. Key components include State Party Annual Reporting (SPAR), Joint External Evaluation (JEE), After Action Review (AAR), Intra Action Review (IAR), and Simulation Exercises (SimEx). Moreover, initiatives like One Health, Emergency Care Systems, Safe Hospitals, and Public Health Emergency Operations Centers (PHEOCs) reinforce preparedness and response capacities. Risk communication and community engagement (RCCE) strategies play a pivotal role in disseminating timely information and fostering community resilience. Furthermore, the management of Chemical, Biological, and Radiological (CBRN) incidents remains a priority, necessitating collaboration between the public health and security sectors. This comprehensive approach aims to strengthen health systems, reduce risks, and improve emergency response capabilities throughout the EMR, thereby promoting global health security and resilience.

**Conclusion:**

The EMR is addressing public health challenges through frameworks like IHR-MEF, and RCCE. It is strengthening emergency care systems, ensuring safe hospitals, and establishing PHEOCs. Proactive measures to address CBRN events and collaboration are enhancing resilience. The inclusion of the One Health approach underscores the EMR’s holistic strategy to address the health threats at the human-animal-environment interface. This demonstrates the EMR’s commitment to global health security.

## Introduction

### Overview of the EMR and its public health threats

The Eastern Mediterranean Region (EMR) encompasses over 500 million people across 22 diverse countries: Afghanistan, Bahrain, Djibouti, Egypt, Iraq, Iran (Islamic Republic of), Jordan, Kuwait, Lebanon, Libya, Morocco, Oman, Pakistan, Palestine, Qatar, Saudi Arabia, Somalia, Sudan, Syrian Arab Republic, Tunisia, United Arab Emirates, and Yemen. These nations vary in their gross domestic products, socio-demographic profiles, health indicators, and health system capacities and coverage (1). Today, the world confronts significant health challenges affecting people, animals, and the environment. Previously, experts addressed these issues separately, but now it is recognized that these health aspects are interconnected and require a collective approach.

In the context of global health emergencies, the WHO-EMR is at a critical juncture. The enormous challenges of public health threats posed by biological, chemical, man-made, and environmental hazards necessitate powerful and proactive response systems based on adequate preparedness and health security in the region. Zoonotic diseases account for 75% of new and emerging infectious diseases in humans, posing major public health threats as seen with COVID-19, Ebola, and MERS-CoV. The Eastern Mediterranean Region faces risks from these and other emerging zoonoses, such as the recent monkeypox outbreak. Dengue fever, Crimean-Congo hemorrhagic fever (CCHF), and avian influenza continue to pose severe health risks with high mortality rates and economic impacts. Human brucellosis is also endemic in many regional countries (2).

The area is also plagued by conflict and civil unrest in Iraq, Syria, Libya, and Yemen. On 15 April 2023, violent clashes erupted in Sudan, resulting in the displacement of over 8.6 million people, including internally displaced persons (IDPs), asylum seekers, and refugees. This conflict has exacerbated many of Sudan’s existing challenges, such as ongoing conflicts, disease outbreaks, economic and political instability, and climate emergencies. Since 7 October 2023, the ongoing crisis between Israeli armed forces and Hamas in the occupied Palestinian territories has led to numerous civilian deaths and injuries. In Gaza, airstrikes and severe shortages of medical supplies have critically strained the health system. The WHO is providing life-saving supplies to address these urgent health needs. With the WHO’s endorsement of a risk-based approach to health emergency management, the EMR is taking various measures to address all these challenges.

### Objectives of the manuscript

This paper looks at the many initiatives and tactics utilized to strengthen the EMR’s preparedness to respond appropriately to existing and upcoming health emergencies. It emphasizes the critical function of strategic risk assessment and the creation of health risk profiles as the first steps toward building a resilient health system. By combining the various strands of emergency care systems, the International Health Regulations, 2005 (IHR) Monitoring and Evaluation Framework (MEF), and the One Health approach, this narrative provides a comprehensive overview of the efforts made to protect public health and ensure a timely, coordinated response to emergencies in the region.

The main objective of this manuscript is to elucidate the comprehensive strategies and learning interventions implemented in the EMR of the WHO to improve preparedness for health emergencies, with a focus on the integration of the IHR 2005 into the regional health security architecture, highlighting its role in building capacity, identifying gaps and facilitating continuous improvement in emergency response.

## Regional capacity strengthening

### Strategic risk assessment and developing and updating health risk profiles in the eastern Mediterranean region (EMR) (Table 1)

**Table 1:** Summary of Regional Emergency Preparedness Strategies and interventions in the EMR.

Strategy/intervention	Purpose	Initiatives
Strategic Risk Assessment and Health Risk Profiles	Identify and prioritize health risks to enhance preparedness	WHO Strategic Tool for Assessing Risks (STAR) - WHO
International Health Regulations (2005) Monitoring and Evaluation Framework (IHR-MEF)	Strengthen health security through core capacity building	State Party Annual Reporting (SPAR) - WHO Joint External Evaluation (JEE) - WHO After Action and Intra Action Reviews (AAR/IAR) - WHO Simulation Exercises (SimEx) - WHO
Points of Entry and Border Health trainings	Enhance capacities at points of entry for rapid response	Member States, IHR (2005) core capacity building training at PoEs - WHO Ship Sanitation inspections and certification training - WHO
One Health Approach	Address zoonotic diseases through multisectoral collaboration	Joint Risk Assessment Operational Tool (JRA OT) - WHO, WOAH, FAO, UNEP
Emergency Care Systems	Improve timely response to acute illnesses and injuries	Emergency Care Framework - WHO Integrated Inter-Agency Triage tool (IITT) - WHO Basic Emergency Care Course (BEC) - WHO, ICRC
Hospital Safety and Hospital Resilience	Ensure hospitals continue functioning during emergencies	Hospital Safety Index Tool - WHO
Public Health Emergency Operations Centers (PHEOC)	Coordinate public health emergency responses	PHEOC Guidance - WHO
Risk Communication and Community Engagement (RCCE)	Strengthen community preparedness and response	Social listening taxonomies - WHO RCCE trainings - WHO
Risk Management of Chemical, Biological, and Radiological (CBRN) Events	Manage risks from CBRN events	Regional Strategic Plan with clear strategic objectives: Advocate for the Health Security Interface (HSI) function, including CBRN - WHO, UNODA, BWC, ISU, UNICRI, OPCW, INTERPOL

WHO, World Health Organization, EMR, Eastern Mediterranean Region, IHR, International Health Regulations, RCCE, Risk Communication and Community Engagement, PHEOC, Public Health Emergency Operations Center, CBRN, Chemical, Biological, Radiological, Nuclear, HSI, Hospital Safety Index, BEC, Basic Emergency Care, UHC, Universal Health Coverage.

The connection between public health risks and emergencies is recognized as arising from the interaction of biological, technological, social, and natural hazards within communities, depending on factors such as likelihood, severity, vulnerabilities, and existing coping capacities. Identifying and assessing hazards and associated risks are critical components of effective emergency management. The World Health Organization (WHO) adopts a risk-based approach to managing health emergencies and mitigating risks, by using an all-hazards approach to emergency management in which health risk management functions are consistent across various types of hazards (3).

To reduce the risks associated with emergencies, WHO-EMRO works closely with countries to develop and regularly update their national health risk profiles, strengthening preparedness and response capacities for identified current and anticipated hazards. WHO EMRO helps countries use the WHO Strategic Toolkit for Risk Assessment (STAR), which allows countries and regions to conduct a strategic, rapid and evidence-based assessment of public health risks to plan and prioritize risk management activities. The assessment results in a country’s risk profile, defining priority hazards and risk calendars where possible (predicting the timings/seasonality of hazards) (4) (Table 2).

**Table 2:** Processes for Defining Global Public Health Emergency Preparedness Strategies and assessing their impact.

Process for defining need (Strategies and interventions)	Process for assessing impact	Examples
Risk Assessment and Profiling: identifying hazards, assessing risks, and prioritizing actions based on likelihood, severity, and vulnerabilities.	Monitoring and Evaluation: Using tools like SPAR, JEE, AAR/IAR, and SimEx to assess preparedness, identify gaps, and improve response capabilities.	- Development of national health risk profiles using WHO’s Strategic Toolkit for Risk Assessment (STAR).
Implementation of IHR Monitoring and Evaluation Framework (MEF): ensuring core capacities are met through continuous learning and improvement.	Feedback Mechanisms: Annual reporting, joint evaluations, after-action reviews to identify strengths and weaknesses.	- Use of SPAR for annual evaluations and JEE for comprehensive evaluations in EMR countries.
Capacity Building: enhancing skills and knowledge through training and exercises.	Performance Metrics: Tracking progress in emergency response readiness and effectiveness.	- Basic Emergency Care Course (BEC) to train healthcare providers in acute care management.
Integration of One Health Approach: addressing zoonotic diseases through multisectoral collaboration.	Cross-Sectoral Coordination: Ensuring alignment and cooperation across health, agriculture, and environmental sectors.	- Joint Risk Assessment Operational Tool (JRA OT) for assessing risks at the human-animal-environment interface.
Hospital Resilience and Safety: strengthening health facility preparedness and response capabilities.	Hospital Safety Index (HSI): Assessing hospital readiness to withstand emergencies.	- Use of HSI tool to evaluate hospital safety and resilience in disaster-prone areas.

SPAR, State Party Annual Report; JEE, Joint External Evaluation; AAR/IAR, After-Action Review / Integrated Action Report; SimEx, Simulation Exercise; IHR, International Health Regulations; EMR, Eastern Mediterranean Region; WHO, World Health Organization.

The hazards prioritization enables rational and effective planning, as well as the most efficient use of limited resources, to improve health emergencies and disaster risk management capacity in the face of multiple and competing priorities. WHO-EMRO efforts are focused on improving health planners’ strategic risk assessment skills and implementing a risk-based approach to emergency planning. This allows countries to develop risk mitigation plans, establish and maintain early warning systems, and initiate and promote multisectoral coordination preparedness and response mechanisms prior to emergencies (5).

### International health regulations (2005) monitoring and evaluation framework

The IHR-MEF, 2005 is critical to improving health security in the EMR by ensuring a well-coordinated and responsive approach to public health emergencies. One of the key components of the IHRMEF is its emphasis on learning interventions and training, which are critical for developing and maintaining the capacity of regional Member States to manage health emergencies and crises. The IHR-MEF encourages continuous development of skills and knowledge among healthcare workers and emergency responders, ensuring they are well-prepared to deal with a wide range of health threats (6).

Through systematic assessment and feedback mechanisms, the framework supports the identification of gaps in core capacities, knowledge, and skills, through different tools including the State Party Annual Reporting (SPAR), the Joint External Evaluation (JEE), the After Action and Intra Action Review (AAR/IAR), and the Simulation Exercises (SimEx), enabling targeted interventions that enhance the IHR core capacities and the regional workforce’s ability to respond to emergencies efficiently and effectively (7–10).

The assessment tools and exercises used enable Member States to identify specific gaps in the health system and develop a National Action Plan for Health Security (NAPHS), by providing the necessary recommendations and standards for preparedness and response, as well as ensuring that countries have access to the best practices and most recent knowledge in the field of public health emergency preparedness. This includes areas such as risk assessment and profiling, health emergency preparedness, One Health, risk communication and community engagement (RCCE), and the implementation of health measures that respect people’s dignity, human rights, and equity. As a result, Member States are better equipped to prevent, detect, assess, notify, and respond to health emergencies quickly, reducing the possible impact on public health and international trade and travel (11). During the final round of SPAR 2023, all Member States (22 countries) completed their annual evaluation; up until January 2024, 20 EMR Member States had completed the first round of JEE, with two countries completing the second round in 2023. AAR has been conducted in six countries in the region in response to various events such as infectious disease outbreaks, natural disasters, and chemical events, whereas IAR was used by 12 countries to review the response to the coronavirus 2019 (COVID-19) pandemic and implement the necessary correction plans. Furthermore, 19 countries used various types of SimEx to review, evaluate and test the procedures, operational plans, guidelines, and standard operating procedures developed to be better prepared to respond to future health threats.

The Universal Health and Preparedness Review (UHPR), which was piloted in Iraq as the second global pilot country, is a significant initiative aimed at improving global health security by encouraging transparency, accountability, and collective action among member states to prepare for and respond to health emergencies. The UHPR is a voluntary peer-review mechanism that encourages the exchange of best practices, challenges, and lessons learned in health emergency preparedness and response. Its significance stems from the promotion of a global culture of health security that transcends borders, emphasizing the interconnectedness of public health and the need for international solidarity. It is critical for identifying gaps, mobilizing resources, and driving improvements in national and international health systems. This proactive approach not only improves the ability to manage and mitigate the impact of health emergencies but also contributes to the achievement of Universal Health Coverage (UHC), ensuring that all people, everywhere, have access to quality health services without financial hardship (12).

Moreover, the IHR-MEF facilitates international cooperation and assistance, leveraging the collective expertise and resources of the global community to support countries in need.

### Points of entry and border health in the EMR

WHO EMRO has been supporting Member States in building the International Health Regulations (2005) core capacity requirements in order to respond to Public Health Emergencies of International Concerns at the designated Point of Entry (PoEs), in different countries, including the airports, ports and ground crossings. This support included regional, sub-regional, and national training programs focused on assessing these capacities at PoEs level. Since the IHR (2005) mandates that Member States should designate PoEs that shall develop the IHR capacities provided according to Annex 1 of IHR (2005), the provided trainings have been centered on conducting risk assessments at PoEs level, designating the PoEs, and engaging the PoEs in simulation exercises to evaluate the trained capacities.

Additionally, specialized training has been provided on ship sanitation inspections and certification, along with vector surveillance and control, food safety, inspection, and environmental health considerations at PoEs. Training sessions specific to event management on aircraft and ships have been rolled out, alongside the development of public health emergency contingency plans and Standard Operating Procedures (SoPs) for use during emergencies (13).

In response to COVID-19, Ebola, and other PHEIC, risk assessment training was conducted to develop travel risk mitigation measures in the context of international travel including guidelines on screening, quarantine, vaccination, and other travel-related measures, in line with WHO recommendations. At the regional level, an important development has been the creation of tailored training packages by WHO-EMR-Office. These packages come in introductory, intermediate, and advanced levels and aim to build the capacities of various stakeholders operating at PoEs (both health and non-health) and guide the development of a national training curriculum. The materials are piloted in several countries and country case studies are documented to reinforce the practical understanding and implementation of the presented technical concepts (13).

### One health

Emerging and reemerging infectious diseases, especially those originating from animals (zoonotic diseases), are a persistent global health threat. Addressing these challenges requires a comprehensive, multisectoral approach, exemplified by One Health (14). WHO-EMR Office in close collaboration with regional and subregional offices of the Food and Agriculture Organization of the United Nations (FAO), the World Organization for Animal Health (WOAH), and the United Nations Environment Program (UNEP) has been collaborating to advance the implementation of On Health and support member states to undertake multiple initiatives, and activities that enhance countries preparedness and efficiently respond for health threats at the human-animal-environment interface (15, 16).

One of the initiatives is to enable professional staff from human, animal, and environmental health authorities to jointly assess the risk of those threats, estimate its likelihood and consequences ahead of time based on existing epidemiological data and expert opinion, and end up with key science-based recommendations and communication messages using Joint Risk Assessment Operational Tool (JRAOT) (17). Several countries have been capacitated to perform this assessment, including Pakistan, Afghanistan, Egypt, Sudan, Qatar, Tunisia, Libya, Iraq, and the UAE. Following the building of capacity at the national level, the national staff in the EMR countries have appreciated the importance of this exercise and the usefulness of the JRAOT in their work, so they have taken this forward through different means, such as organizing a cascade training for professional staff at the province level as in Egypt while other countries have used this exercise to assess the risk of zoonotic diseases spread and its related consequences before celebrating large events and mass gatherings in the country as assessing middle east respiratory syndrome coronavirus (MERS-COV), Brucellosis and HPAI in Qatar before the FIFA World Cup.

### Emergency care system: emergency care toolkit, basic emergency care course

Well-developed emergency care systems provide timely recognition of urgent conditions, resuscitation and referral for severely ill patients, and the delivery of definitive care for a range of acute illnesses and injuries in both children and adults. Emergency care is an essential part of UHC. Moreover, emergency care systems are indispensable to achieving Sustainable Development Goal (SDG) targets in maternal and child health, non-communicable and infectious diseases, disasters, injuries, and violence. The challenges of the COVID-19 pandemic, climate change, urbanization, and recognition of the need to improve the quality and efficiency of health services have led Ministries of Health to focus on developing effective emergency care systems over the last decade. Recognizing that the effectiveness of many proven health interventions is reduced when care is delayed, the Member States of the World Health Assembly passed a resolution in 2023 on Integrated emergency, critical, and operative care for UHC and protection from health emergencies, recognizing that emergency, critical, and operative care services are necessary to execute the core capacities under the IHR (2005) and to promote the enjoyment of human rights.

The WHO Emergency Care Framework, developed by WHO, describes the essential functions and components of an effective emergency care system – from where the emergency occurs, through prehospital care and transport, to the emergency unit, and on to definitive care (18). Additionally, an Emergency Care Toolkit was developed which is a range of clinical process guidance tools that can be implemented in facilities to ensure that life-threatening conditions are not missed and that timely life-saving interventions are performed. This includes; the integrated inter-agency triage tool (IITT) to categorize and prioritize patients arriving at the emergency room, the Emergency Care Checklists for trauma and medical emergency cases management, Standardized Clinical Forms for trauma and medical emergency cases to ensure proper patient registry and data collection, the Resuscitation Area Designation (Checklist) to ensure optimal availability of staff, space, and stuff to treat critically ill and injured patients in emergency units and finally the Basic Emergency Care Course (BEC) (19).

Developed by WHO and ICRC, in collaboration with the International Federation for Emergency Medicine, Basic Emergency Care (BEC): Approach to the acutely ill and injured is a training course for front-line healthcare providers who manage acute illness and injury with limited resources. BEC teaches a systematic approach to the initial assessment and management of time-sensitive conditions where early intervention saves lives. The course trains participants to be prepared to deal with a variety of critical illnesses, with a focus on trauma, breathing, shock, and altered mental status. The BEC course is intended for individuals who might be able or expected to provide emergent patient care, including students, trainees, nurses, physicians, and even prehospital or inpatient care providers, among others. This course is not only intended for emergency medicine physicians, but for all types of locally appropriate providers. The Methodology of training is comprised of didactic lectures, Interactive workbook questions, Case scenarios and hands-on skills sessions (19).

The WHO-EMR Office recognizes the regional need to support the clinical processes in the emergency units and improve the clinical capacity of front-line healthcare providers to deal with the complex emergencies happening in the EMR that comprise a wide range of events from natural disasters to conflicts, climate change induced emergencies, and other manmade disasters. The support provided to countries includes training the concerned nationals on the different components of the emergency care toolkit with a focus on the basic emergency care course. The goal is to scale up emergency care capacities in countries with the main goal of improving emergency conditions outcomes and decreasing mortality and morbidity by providing participants with the knowledge and skills to practice a systematic approach to the initial assessment and management of time-sensitive conditions where early intervention saves lives (20).

### Safe, emergency and disaster resilient hospitals: hospital safety index tool

Developing safe and disaster-resilient health facilities and hospitals has been a major focus of support provided by the WHO-EMR Office with various training and technical assistance extended to the member states in the region. This initiative aims to develop the capacity of hospitals to manage emergencies and disasters and to continue providing health services to the affected population even after the impact of a danger. To strengthen hospital resilience in countries, WHO-EMR Office supports member states in developing their capacity to identify and better understand the vulnerabilities of hospitals that can potentially hinder their operations during emergencies and to strengthen capacities across the different levels of the disaster management cycle while pooling in all existing resources and tools for operationalization. By doing so, hospitals will be able to integrate improvement actions into their development plans to reduce disaster risks and be better prepared to respond to emergencies and develop disaster resilience.

The Hospital Safety Index tool, developed by WHO, provides a snapshot of the probability that a hospital or health facility will continue to function in emergencies, based on structural, non-structural, and functional factors, including the environment and the health services network to which it belongs. By determining a hospital’s safety index or score, countries and decision-makers will have a general idea of their ability to respond to major emergencies and disasters and will be able to improve capacities when needed (21). In this connection, the CPI unit in the WHO-EMR Office trains national teams of evaluators in countries on the hospital resilience concept and its operational resources, with a focus on the Hospital Safety Index tool along with hands-on training on its application using information from existing hospitals. This step is important to provide an opportunity for the teams to build on this initiative and apply the Health Security Interface (HIS) Tool by learning how to conduct the assessment in their respective countries.

### Public health emergency operations center

EMR Office has been working to strengthen the emergency preparedness and readiness capacities of the country to support a well-planned and coordinated emergency response. The WHO as a leading public health agency globally adopts the PHEOC concept and develops PHEOC guidance for public health, building on other sectors’ successes and lessons learned. A functional emergency management system through a well-established PHEOC is crucial for coordinating the prevention, preparedness for, and response to public health emergencies. It is a platform that facilitates the participation of various stakeholders and ensures better management of information and resources before and during response operations. The functionality of PHEOCs in countries is vital to their response capacity. Many countries have established PHEOCs, and some were successful in utilizing them in their COVID-19 response alongside other emergencies (22, 23).

Human resources are one of the most precious and scarce resources in the region in terms of number and mix of skills. Strengthening national capacities on the role and functionality of the PHEOC for emergency management is one of the modalities. Staff working in PHEOC need well-defined Terms of Reference (ToRs) clear works SOPs and a regular training program that equips them with the right competencies to perform their duties (24). Staff should not be assigned to roles and responsibilities unknown to them: The PHEOC roles must be aligned as closely as possible with their established skill sets. Staff should receive thorough orientation in a PHEOC setting and training specific to the functions, roles, and procedures they will undertake therein. A training needs assessment – either at the organizational/institutional level or for individuals—proceeds from an assessment of the knowledge, skills, and abilities (competencies) people require to be able to work effectively in a PHEOC, as well as of their training needs and the existing opportunities for collaboration with partners and other sectors. These needs are then compared with known or identified shortfalls to formulate training objectives. The training program is then designed, developed, delivered, evaluated, and projected forward to the next level of training requirements as successive groups of trainees progress from basic awareness to working-level knowledge, and then to advanced competence. Participants in a training program will undertake pre-and-post-training evaluations to confirm that their training objectives have been addressed.

Personnel assigned to work in a PHEOC have three types of training requirements: Training in the incident management system used at the PHEOC, training in the specific function the person is expected to perform within the PHEOC, and emergency management training is part of the subject matter expertise the trainee brings to the operation. There are many recognized training processes to establish knowledge and skills to plan, train, exercise, evaluate, and monitor emergency management. Preparedness phases abilities required to function effectively in a PHEOC staff include the following: Training options include classroom-based courses, e-learning, PHEOC planning and procedure development, site and field assignments for hands-on experience, and team-building exercises. The maintenance program for a PHEOC requires ongoing support from government and private donors in many areas including conducting training and exercises.

### Risk communication and community engagement

RCCE strategies have been instrumental in bolstering support during various health emergencies, as demonstrated by the robust response to crises such as the Syria-Türkiye earthquake, the monkeypox (Mpox) outbreak, and the Sudan conflict. Targeted training packages on crucial health concerns including water, sanitation, and hygiene (WASH), vaccine-preventable diseases, mental health support, and more were deployed across impacted countries. These interventions, created in partnership with entities such as the International Federation of Red Cross and Red Crescent Societies (IFRC) and the Syrian Arab Red Crescent (SARC), aimed to strengthen community preparedness and streamline access to critical health services. Complementing these efforts, the RCCE employed social listening taxonomies and biweekly reporting systems to capture community insights, thus informing customized RCCE and public health interventions (25).

Subsequent RCCE training in the EMR adopted a comprehensive approach to empower communities. Training for the Mpox response highlighted the necessity to raise awareness and allow community participation in prevention efforts, focusing on stigma reduction and community resilience through tailored messaging. Collaborative efforts with academic institutions expanded the efficacy of health interventions through KAP studies to assess community knowledge, attitudes, and practices (26). Moreover, RCCE leveraged social listening for the strategic development of communication materials targeted at cholera and vector-borne disease risks in the aftermath of the Libya floods, showcasing adaptability amidst diverse health emergencies. Building on the foundation of immediate crisis response, RCCE invested in capacity building for health personnel, particularly evidenced by the tailored three-day training that responded to the surge in meningitis in northwest Syria. The training integrated behavioral insights, risk communication, and community feedback mechanisms, highlighting the necessity of community engagement at all levels of the health response continuum.

On a broader scale, the reinvigoration of the Eastern Mediterranean Region Interagency Working Group (IAWG), initiated initially for COVID-19, attested to RCCE’s far-reaching commitment to capacity building across multiple health emergencies, ensuring consistent messaging and cooperative response to the Mpox and cholera outbreaks, Morocco earthquake, Libya floods, and escalating hostilities in Gaza. This collaborative framework enabled stakeholders to harmoniously share expertise and resources, enhancing emergency response quality and unity (27).

Furthermore, infodemic management training in countries like the Islamic Republic of Iran and Jordan is indicative of a comprehensive educational philosophy aimed at equipping health systems and communities to withstand future crises. The regional social listening system, which uses AI-driven tools and diverse online platforms, was vital for timely and effective risk communication and health messaging across a spectrum of pressing health crises. In parallel, hands-on exercises, such as SocialNet simulation and deployment training, facilitated a rich interregional exchange of knowledge and practices, which are invaluable for the Region’s strengthened response structures. These learning experiences inform crucial insights into RCCE, adaptable to various settings, thus strengthening preparedness and response capacities for health emergencies. The scope of RCCE educational initiatives has played a significant role in the resolution of health crises, underscoring a commitment to capacity building and ongoing community involvement throughout the EMR. These strategic educational efforts have contributed to the strengthening of health systems and supported the well-being of communities in Egypt, Jordan, Lebanon, Libya, Morocco, Pakistan, Sudan, Syria, and Tunisia, improving the regional emergency response framework as it adapts to emerging public health challenges (28).

### Risk management of chemical, biological, and radio nuclear events

Countries in the EMR are frequently affected by various public health emergencies, including disease outbreaks, natural disasters, conflicts, and civil wars. Vulnerability to these risks, *inter alia*, is increased by factors such as low immunization rates in some conflict-affected countries, and high numbers of internally displaced people. Furthermore, full achievement of the public health capacities required under the IHR (2005) to effectively prevent, detect, and rapidly respond to any public health threat remains a work in progress. Although the primary responsibility for the management of the risk of Chemical, Biological, and Radiological (CBRN) events lies with the national governments, WHO is well placed to coordinate specific global public health preparedness and operational readiness, including early warning, threat detection and prioritization, risk management, and global surveillance. In addition, WHO can support Member States in epidemiological investigations and health consequence management of CBRN, in collaboration with key partners in international law enforcement and security domains. A regional risk assessment was conducted in the EMR using the WHO strategic tool for assessing risks (STAR) to identify potential natural and human-induced hazards in the Region. Twenty-three risks were identified as potential hazards that currently or may potentially require regional intervention, of which CBRN events were identified as one of the three highest risks, alongside armed conflict and forced population displacement.

The EMR Office developed a health and security function and initiated a draft Regional Strategic Plan with clear strategic objectives: Advocate for the HSI function, including CBRN, improve EMR capacities for country preparedness for CBRN events, and promote partnerships to support public health and security in the EMR. The implementation of public health work, in coordination with the security sector, increased the effectiveness of preparedness and response to global health security risks. New challenges to public health are being faced worldwide in the form of increasingly and easily accessible dual-use technologies, and growing disease burdens from emerging and reemerging pathogens due to the effects of climate change, in combination with increased mobility. These are compounded by population growth due to displacement, and an increasing frequency of terrorist incidents and perceived terrorist threats in the EMR and neighboring regions, among other challenges. Most of the HSI activities have been implemented in partnership with key partners (such as UNODA/ BWC/ ISU, UNICRI, OPCW, INTERPOL and WHO sister units from different specialties) with objectives to enhance EMR member states capacities for country preparedness for CBRN events and promote partnerships to support public health and security sectors (29).

## Lesson learned from the implementation of the emergency preparedness strategies and interventions in the EMR

Several critical lessons have been learned from the adoption of emergency preparedness methods and interventions in the emergency management system. Comprehensive evaluations, particularly the JEE, have been crucial in showing strengths and flagging critical gaps in preparedness among EMR countries. These assessments highlight the importance of identifying baseline preparation levels and prioritizing improvements. Capacity-building initiatives, such as the BEC, have proven critical in providing healthcare workers with the skills required to efficiently handle acute care during crises.

Multisectoral collaboration, as illustrated by the One Health Approach, has proven important in combating zoonotic diseases and health concerns at the human-animal-environment interface. This strategy emphasizes the necessity of coordinating efforts in the health, agriculture, and environmental sectors. Feedback methods, such as SPAR and AARs, have supplied useful information about emergency response systems’ strengths and deficiencies. The continuous review and modification of tactics is crucial for improving reaction capabilities over time.

Adaptation to local contexts has been critical, with distinct cultural, socioeconomic, and geographical factors determining response efficacy in various EMR regions. Assessing hospital resilience using instruments like the HSI has highlighted the significance of ensuring that healthcare facilities can resist and respond to emergencies successfully. Integrating technical breakthroughs and innovations, such as digital health and telemedicine, has improved the efficiency and effectiveness of emergency response activities.

The lessons learned from implementing emergency preparation in the EMR will shape future initiatives, stressing comprehensive capacities assessments like JEE, focused capacity building like BEC training, and multisectoral collaboration via the One Health Approach. Feedback mechanisms and local flexibility will improve reaction frameworks, whereas incorporating digital health will improve communication and healthcare delivery during crises. Lessons in hospital resilience from technologies like HSI will strengthen healthcare facilities and ensure effective crisis management. These findings aim to increase readiness, collaboration, and adaptable tactics for future emergencies in the region.

The implications of lessons learned for further emergency preparedness research in the EMR underscore the need for targeted research in the EMR to improve emergency preparedness. This includes continuous refining of different assessment tools like the SAPR, JEE, and AAR, assessing the efficacy of capacity-building efforts like BEC training, investigating the integration of digital health technology, and researching hospital resilience using tools like the HSI. These research activities are critical for developing future disaster preparation plans and interventions in the region, ensuring that they are evidence-based and responsive to changing health concerns and dangers.

## Global capacity strengthening

The International Health Regulations and One Health approach play a pivotal role in integrating national and global preparedness measures, ensuring a cohesive and comprehensive strategy for managing health emergencies. The IHR, adopted by WHO State Parties, is designed to prevent, protect against, control, and provide a public health response to the international spread of disease. It mandates countries to develop core capacities to detect, assess, report, and respond to public health threats, thereby strengthening internal preparedness. In addition, the IHR fosters international collaboration and transparency through IHR MEF including, SPAR, JEE, and AAR (7–10). These tools help countries identify gaps in their health systems and implement targeted interventions, which are crucial for aligning national preparedness efforts with global health security objectives. By participating in these evaluations and reporting systems, countries enhance their ability to manage health threats internally while contributing to a coordinated international response.

The One Health approach, which recognizes the interconnectedness of human, animal, and environmental health, further integrates internal and external preparedness measures. This approach promotes multisectoral collaboration among various stakeholders, including health, agriculture, and environmental sectors, to address zoonotic diseases and other health threats that arise at the human-animal-environment interface. By implementing Joint Risk Assessment Operational Tools (JRA OT) and other collaborative frameworks, countries can assess and manage risks more effectively, enhancing their national capacity to respond to health emergencies. Simultaneously, the One Health approach facilitates global cooperation and knowledge sharing, enabling countries to benefit from global expertise and resources. This dual focus on internal capacity building and international collaboration ensures a holistic and robust preparedness strategy, capable of addressing complex and evolving health challenges.

## Conclusion

The EMR confronts a wide range of evolving public health challenges, necessitating comprehensive preparedness and response strategies. Countries in the EMR are improving their ability to manage health emergencies through tools and frameworks such as STAR and the IHR-MEF, as well as initiatives such as RCCE. Additionally, efforts to strengthen emergency care systems, ensure safe hospitals, and establish PHEOCs demonstrate a commitment to increasing the resilience of the health system. Furthermore, proactive measures to address CBRN events demonstrate the region’s commitment to mitigate emerging threats. By encouraging collaboration, capacity building, and knowledge sharing, the EMR is well-positioned to navigate future health crises with agility and resilience. This collaborative effort highlights the importance of global health security and the EMR’s commitment to protecting public health and well-being in the face of adversity.
